# Supplementary material for: A moonlighting role for enzymes of glycolysis in the co-localization of mitochondria and chloroplasts
Source: Nat Commun. 2020 Sep 9;11:4509. doi: 10.1038/s41467-020-18234-w (PMC7481185; doi:10.1038/s41467-020-18234-w)
Supplement: Supplementary file 1 — Supplementary Information [file 41467_2020_18234_MOESM1_ESM.docx]

### Supplementary Information

### Supplementary note:

**Complementation of the morphological and cell biological phenotypes**

The physical interaction between mitochondria and chloroplast appears to be greatly influenced by the phosphoglycerate mutase-enolase-pyruvate kinase association which we demonstrate in the section of result part has the capacity to very highly efficiently convert 3PGA to pyruvate. In order to further study the importance of the constituent enzymes in the co-localization of mitochondria and chloroplasts we studied the phosphoglycerate mutase double mutant and various complemented versions of these mutants at the morphological and cell biological levels. The mutants could be fully complemented at both the enzyme activity and plant morphology levels following the expression of the corresponding gene under the control of its native promoter ^1, 2^. Furthermore, the nuclear sublocalized enolase could complement the enzyme activity without complementing the glycolytic metabolon, while it could neither complement the plant growth phenotype ^1, 3^, nor the cell biological phenotype (Figures 4E and 5F). In addition we alternatively attempted complementing the *pgam* double mutant with the full length PGAM targeted to the nucleus, with a site-directed-mutant effecting a residue of the active site of PGAM1 and thus being catalytically inactive ^2^, or with the *E. coli* PGAM (Supplementary Figure 7). In the nuclear sublocalized PGAM1 complementation lines, the enzyme activity could be partial complemented to levels resembling those previously reported for the single *pgam* mutants ^2^ while the plant growth and development could not be with the complemented lines still produced less seeds (Supplementary Figure 8A). In addition, the side-directed-mutated Arabidopsis PGAM could neither complement the enzyme activity nor the plant growth and developmental phenotypes (Supplementary Figure 7). The *E.coli* PGAM did not interacted with enolase or TPT(Supplementary Figure 2) and could only recover 50% enzyme activity (Supplementary Figure 8B), and it also neither complemented seed production or seed growth (Supplementary Figures 7 and 8).

As stated above the double *pgam* mutants were characterized as displaying a dramatically reduced association of mitochondria to the chloroplast, we thus next analyzed this trait in the complemented lines. In the lines complemented by the site-directed-mutant of the Arabidopsis PGAM, the cell biological phenotypes could be complemented (Figures 6C, E and Supplementary figure 3C) Given that expression of the *E.coli* PGAM strongly effected seed production and seed growth (Supplementary Figure 8), we were unable to analyze its cell biological phenotype, whilst as mentioned above the nuclear targeted PGAM complementation line could not complement the mitochondria association (Figure 5E), but could complement the mitochondria displacement (Figure 6F). Thus under optimal growth conditions it would appear that the activity of this glycolytic enzyme is more crucial than its subcellular location for normal growth but that expression of the correct enzyme activity is required for proper fertility.

**Supplementary figures:**


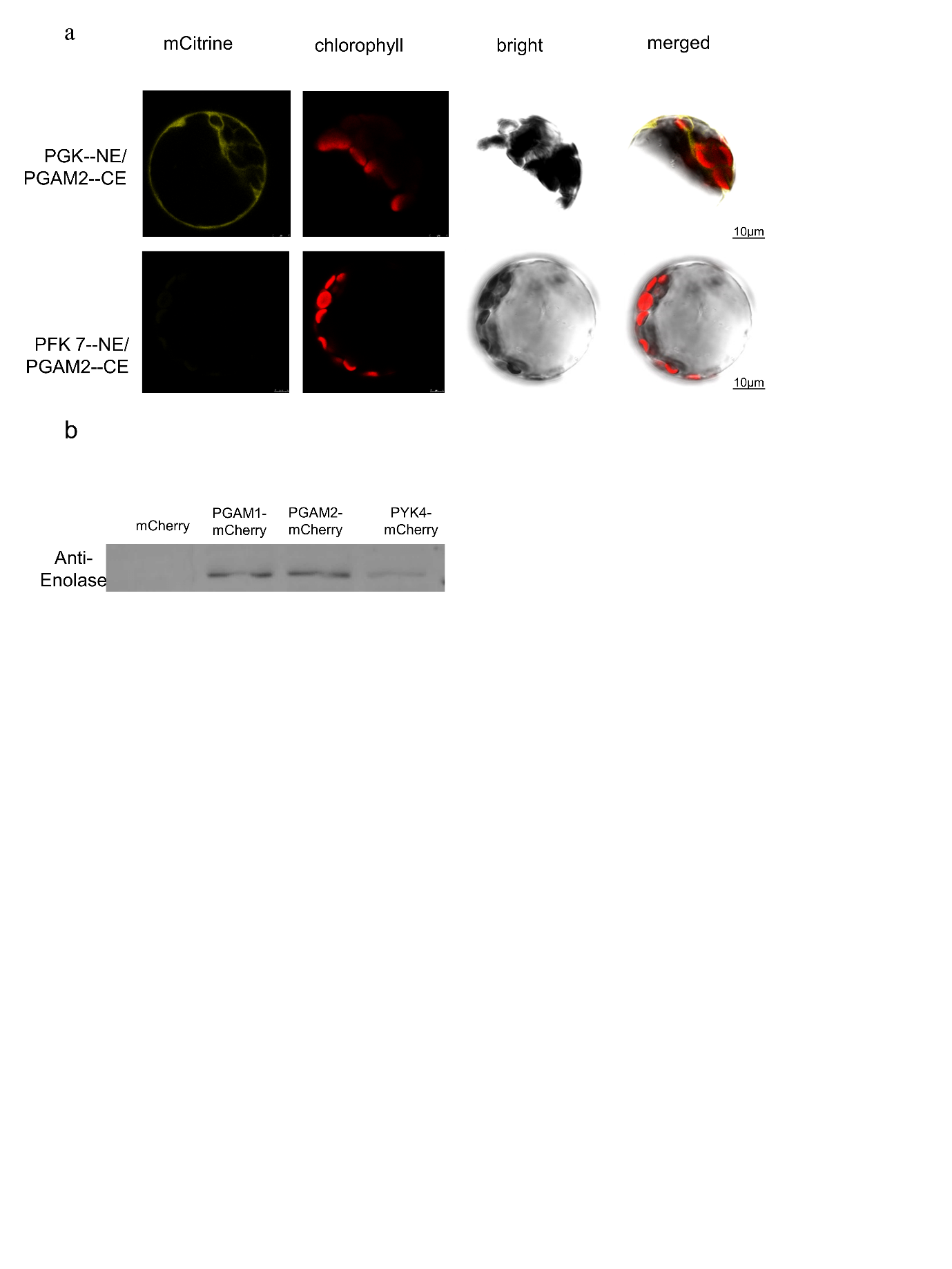


**Supplementary Figure 1. Confirmation of selected protein-protein interactions.** a, The possible interaction of all the glycolytic enzymes were further tested by BiFC with transient expression of tagged proteins in Arabidopsis mesophyll protoplasts and only the detected interaction was presented here. The panels from the left-side show the BiFC fluorescence, fluorescence-CE from bright field image, blank and the merged image of all of those, respectively. The detail of the constructs can be found in method and material. PFK7--NE/ PGAM 2--CE was shown as a representative negative control. b, Confirmation of protein-protein interaction by Co-immunoprecipitation assays. The mCherry tag-based bait and mCitrine tag-based prey were transient expressed in Arabidopsis leaves.


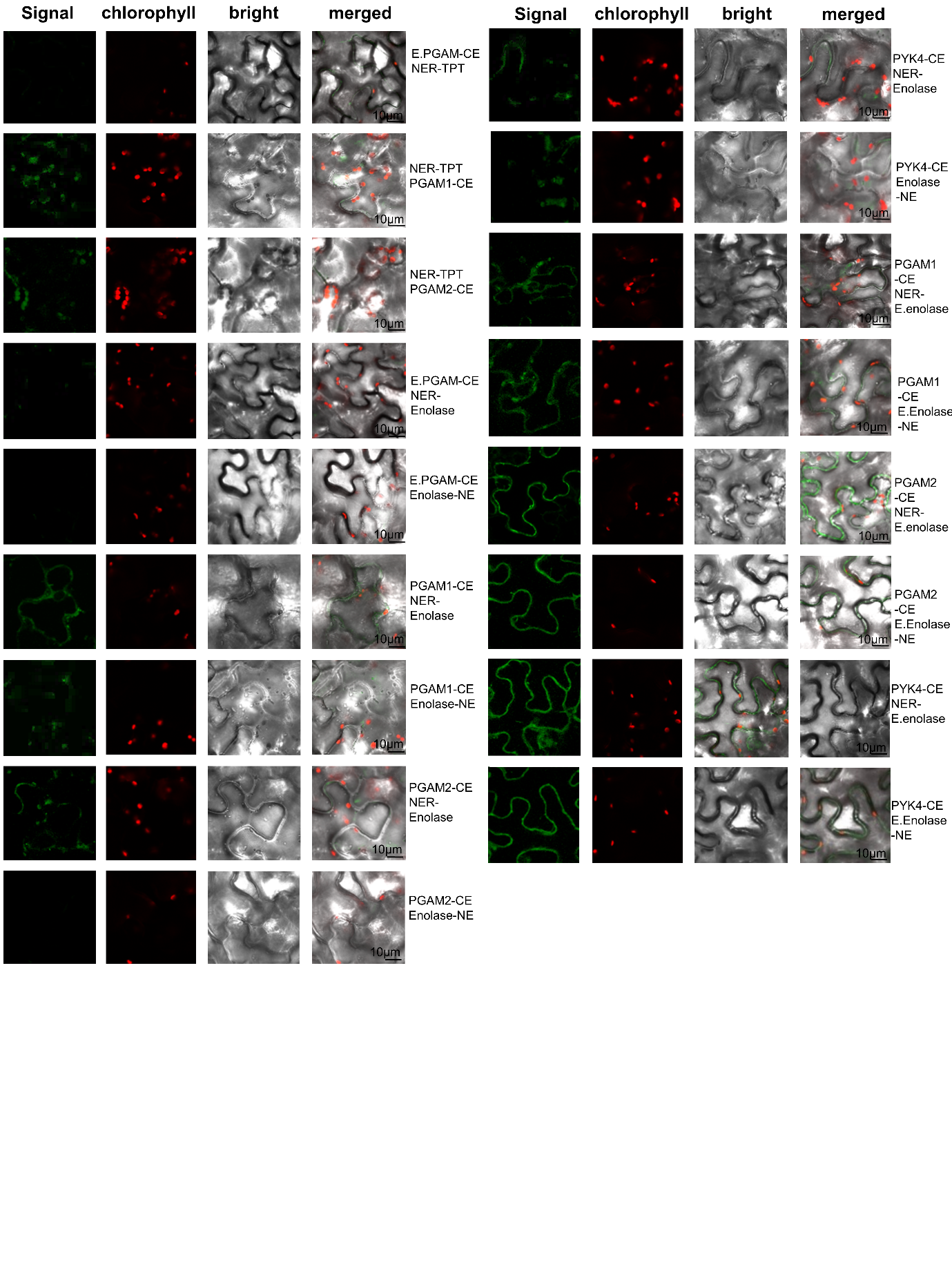
**Supplementary Figure 2. Confirmation of phosphoglycerate mutase 1-Enolase-PYK4 complex with TPT, *E.coli* enolase (E.enolase), *E.coli* (E.PGAM) phosphoglycerate mutase by BiFC assay in Arabidopsis leaves.** The panels from the left-side show the BiFC fluorescence, fluorescence from bright field image, auto fluorescence, blank and the merged image of all of those, respectively. NE is the N-terminal of the split mCitrine, CE is the C-terminal of the split mCitrine.


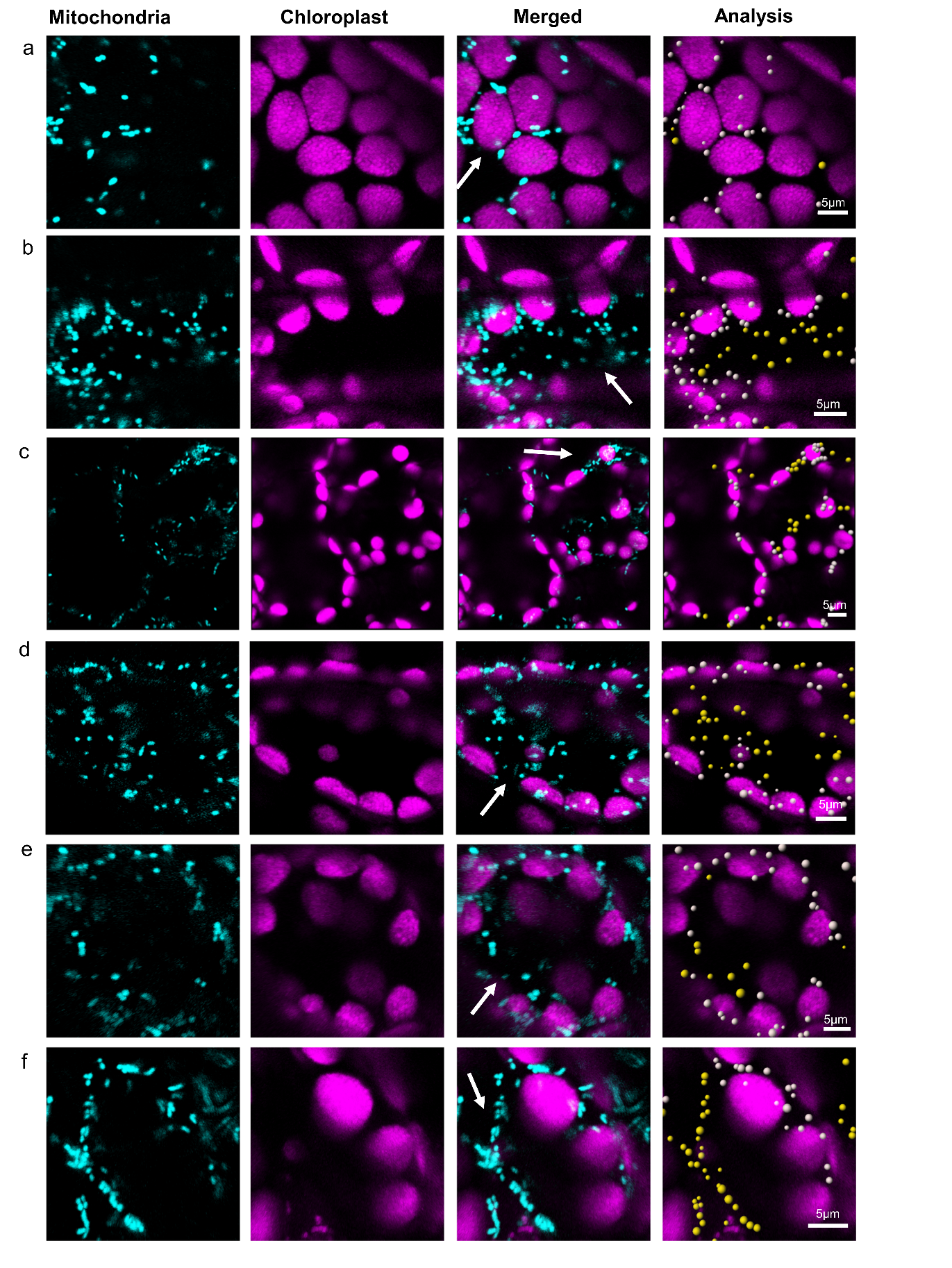


**Supplementary Figure 3. The association of mitochondria and chloroplast.** The mitochondria associated at middle of night (MN) in WT (a), *pgam1/2* (b), *sdmA-pgam1/2* (c), *enolase-2* (d) and enolase-4 (e). The mitochondria associated at middle of day (MD) in enolase-4 (f). *pgam1/2* is the double mutant of *phosphoglycerate mutase 1 and phosphoglycerate mutase. sdmA-pgam1/2* is the double mutant complemented by native promoter with nonfunctional phosphoglycerate mutaseEnolase-2 and enolase-4 are two mutant of enolase. The cyan fluorescence is mitochondria; purple is the auto fluorescence; yellow is represent the un-associated mitochondria and white is represent associated mitochondria respectively. Note: scare bar is different at each figure.


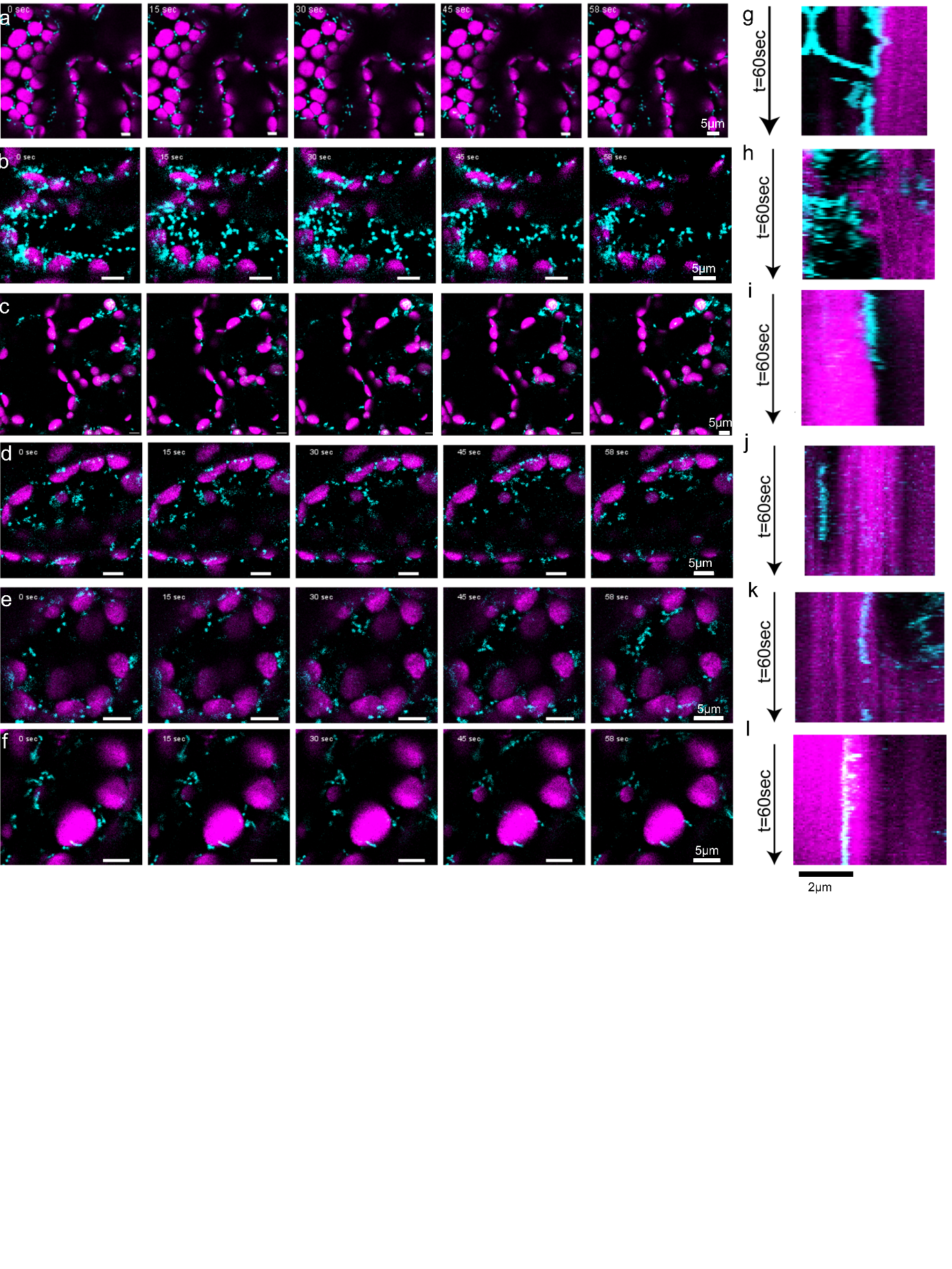
**Supplementary Figure 4 The analysis of mitochondria movement in the plant mature leaves.** A, The mitochondria movement analysis at 1mins at middle of night (MN) in WT (a), *pgam1/2* (b), *sdmA-pgam1/2* (c), *enolase-2* (d) and *enolase-4* (e). The mitochondria movement analyses of *enolase-4* (f) at middle of day (MD). The cyan fluorescence is mitochondria, purple is the auto fluorescence. The mitochondria of WT are move around chloroplast, while the mitochondria are randomly and disorderly moved in the cell. Kymograph of the mitochondria movement at middle of night in WT (g), pgam1/2 (h), pgam1/2-SDM (i), enolase-2 (j), enolase-4 (k) at middle of day in enolase-4 (l). The mitochondria attached to chloroplast at 10 to 15 seconds and move to other place both at day and night in the WT, while there is not attachment between mitochondria and chloroplast in all of the mutant. Note: scare bar is different at each figure.


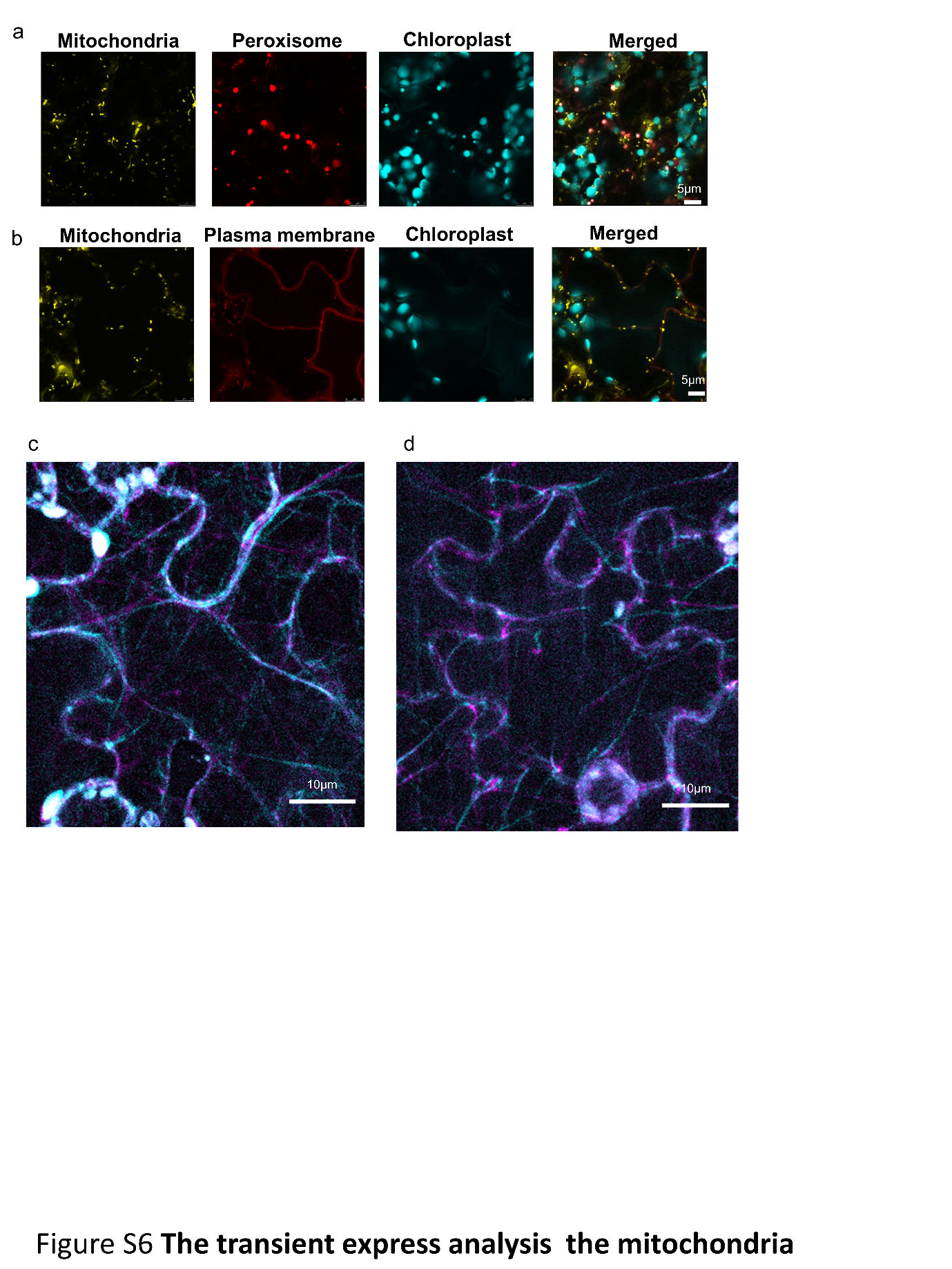
**Supplementary Figure 5 The transient express analysis the mitochondria association with peroxisome and plasma membrane.** a, Both mitochondria and peroxisome markers are transiently co-expressed in Arabidopsis leaves. b, Both mitochondria and plasma membrane markers are transiently co-expressed in Arabidopsis leaves. c, Imaging of actin markers treated with mock at 40mins. d, Imaging of actin markers treated with Enoblock. Images represent a merge of two time points take forty seconds apart (Cyan 0 seconds and Magenta 40 seconds). No drastic changes in actin filament organization or displacement is observed between the mock and enoblock.


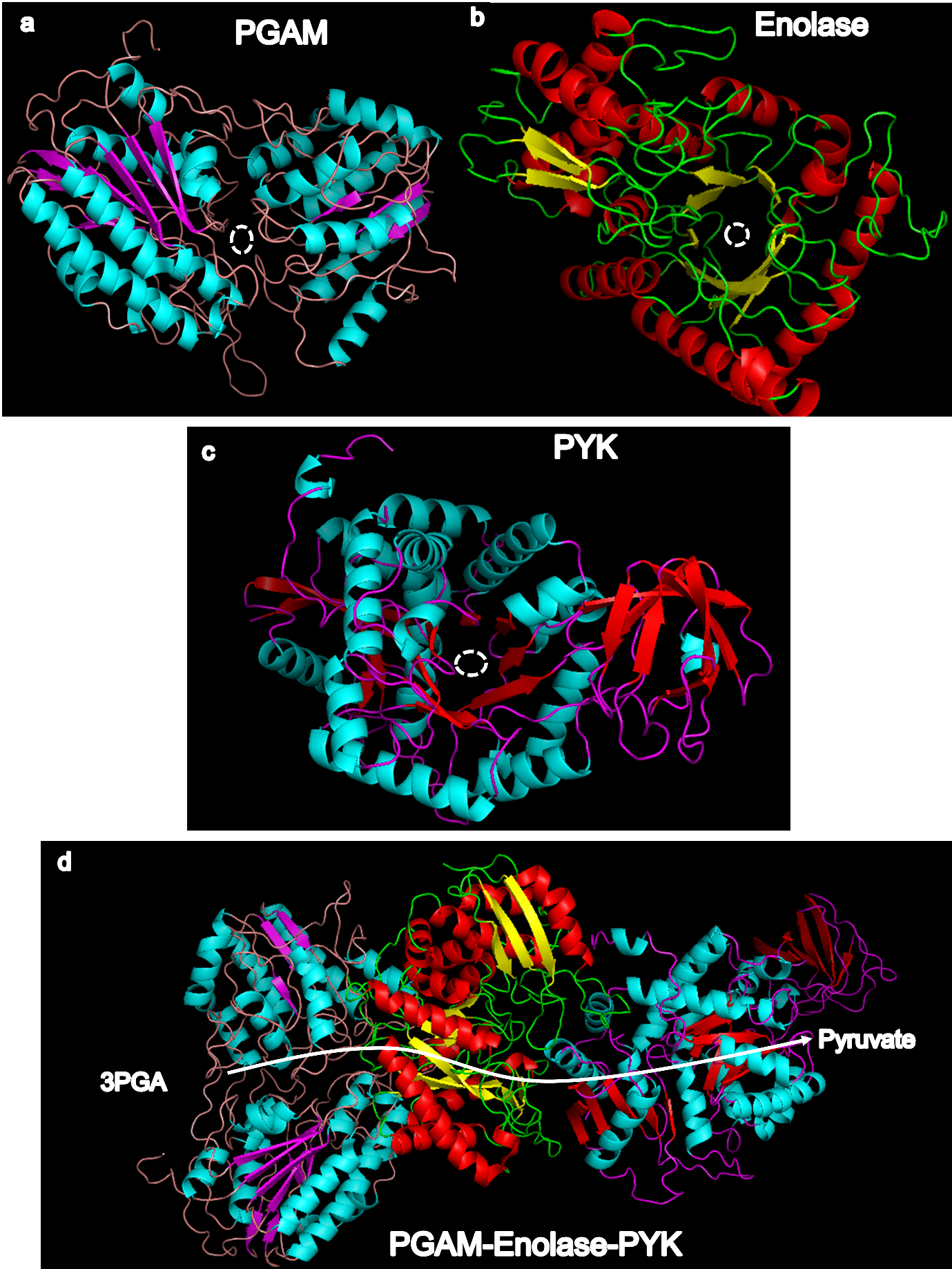
**Supplementary Figure 6 Protein structure of plant phosphoglycerate mutase 1, enolase, PYK4 and complex.** a, Homology modeling structure of phosphoglycerate mutase; b, Homology modeling structure of enolase 2; c, Homology modeling structure PK4. All the structures were produced by the homology modeling software I-TASSER with more than 0.5 identities. d, the docking structure of phosphoglycerate mutase 1-Enolase-PK4 metabolon by Cluspro 2.0.


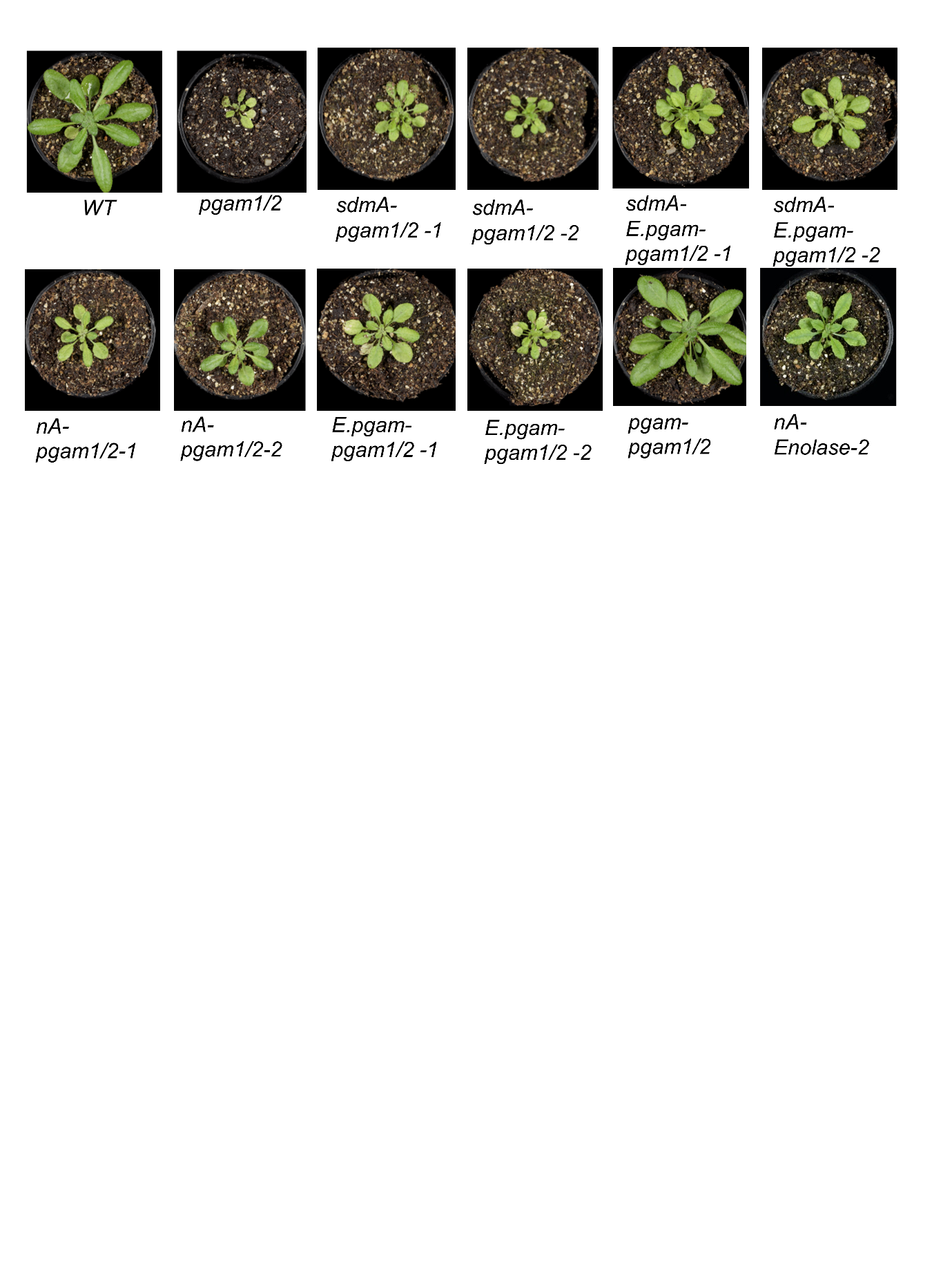
**Supplementary Figure 7. Plant growth phenotype and metabolites profiling of *phosphoglycerate mutase* double mutant complement lines *and WT.*** A, The 35 days plant phenotype in the green house with short day condition (8h light and 16h dark). The double mutant of *phosphoglycerate mutase* is very small in the soil. *nA- pgam1/2-1* and *nA- pgam1/2-2 are* two complementation lines native promoter PGAM1 with nuclear target PGAM1. *sdmA-pgam1/2 -1* and *sdmA-pgam1/2 -2* are two complementation lines native promoter PGAM1 with nonfunctional PGAM1. *E.pgam-pgam1/2 -1* and *E.pgam-pgam1/2 -2* are two complementation lines native promoter enolase with *E.coli* PGAM. *sdmA-E.pgam-pgam1/2 -2* and *sdmA-E.pgam-pgam1/2 -2* are two complementation lines native promoter enolase with *E.coli* PGAM and native promoter PGAM1 with nonfunctional PGAM1. *pgam-pgam1/2* is the complementation lines native promoter PGAM1 and full length PGAM1. *nA- enolase-2* is the complementation line native promoter enolase with nuclear target enolase. All the complemented lines presented growth slowly compared with the wild type and full completed lines. B, Metabolites profiling presented in the heat map calculated by log2 fold change. 36 days old plant leaves of LD condition were collected in the morning and measured the metabolites by GC-MS.


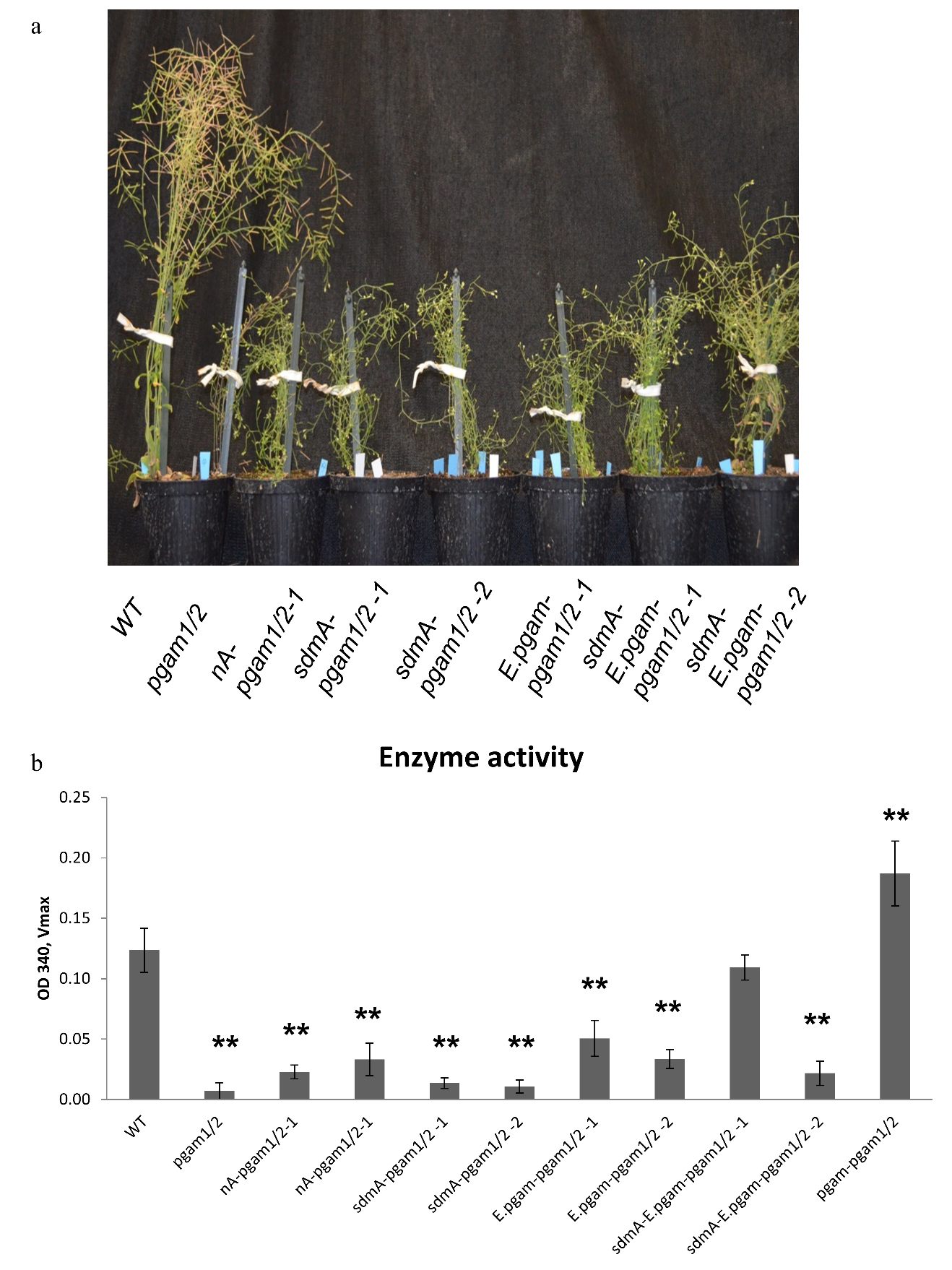


**Supplementary Figure 8 Plant growth phenotype and enzyme activity of *phosphoglycerate mutase* double mutant complement lines *and WT.***  a, Seeds production of the *phosphoglycerate mutase* double mutant complement lines. The nuclear sublocalized PGAM1 complementation lines could produce less seeds. The complementation lines of enzyme activity mutated PGAM have very few seed production. The complementation lines *E.coli* PGAM couldn’t produce any seeds in homozygous. The complementation lines of both *E.coli* PGAM and enzyme activity mutated PGAM also couldn’t produce any seeds. b, The enzyme activity of all the *phosphoglycerate mutase* double mutant complement lines and WT. One way ANOVA analysis by WT control (one star is P<0,05, two star is P<0.01, SD).


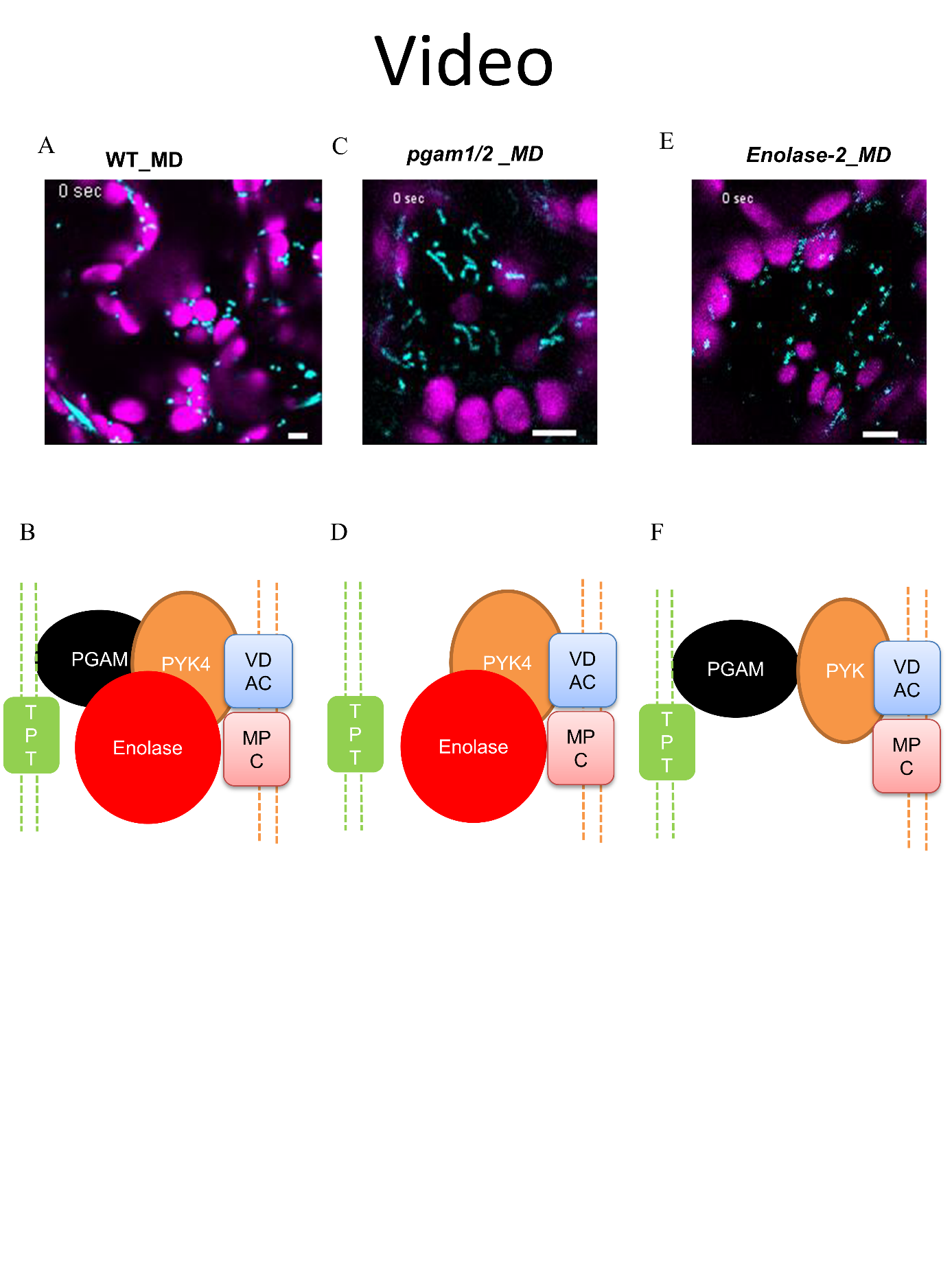


**Supplementary Figure 9 Movie mitochondria movement Movie and complex formation** A, (Movie 1), The mitochondria movement in WT Arabidopsis. Mitochondrial target mCitrine (MTS-mCitrine) was transformed into Wild type col-0 Arabidopsis plants. The cyan fluorescence is mitochondria and purple is the auto fluorescence, respectively. B, The full complex between mitochondria and chloroplast of Movie 1. C (Movie 2), The mitochondria movement in the double mutant of the phosphoglycerate mutase (*pgam1/2*). D, The partly complex between mitochondria and chloroplast of Movie 2. E (Movie 3), The mitochondria movement in mutant the of enolase. F, The uncompleted complex between mitochondria and chloroplast of Movie 3.

Supplementary References:

1. Eremina M, Rozhon W, Yang S, Poppenberger B. ENO2 activity is required for the development and reproductive success of plants, and is feedback‐repressed by AtMBP‐1. *The Plant Journal* **81**, 895-906 (2015).

2. Zhao Z, Assmann SM. The glycolytic enzyme, phosphoglycerate mutase, has critical roles in stomatal movement, vegetative growth, and pollen production in Arabidopsis thaliana. *Journal of experimental botany* **62**, 5179-5189 (2011).

3. Kang M, Abdelmageed H, Lee S, Reichert A, Mysore KS, Allen RD. AtMBP‐1, an alternative translation product of LOS2, affects abscisic acid responses and is modulated by the E3 ubiquitin ligase AtSAP5. *The Plant Journal* **76**, 481-493 (2013).
