# Supplementary material for: A moonlighting role for enzymes of glycolysis in the co-localization of mitochondria and chloroplasts
Source: Nat Commun. 2020 Sep 9;11:4509. doi: 10.1038/s41467-020-18234-w (PMC7481185; doi:10.1038/s41467-020-18234-w)
Supplement: Supplementary file 3 — Descriptions of Additional Supplementary Files [file 41467_2020_18234_MOESM3_ESM.pdf]

Descriptions of Additional Supplementary Files

Supplementary Data 1

Description: List of the proteins tested in this study.

Supplementary Data 2

Description: All the primers used in this study.

Supplementary Data 3

Description: Plasmids constructed in this study.

Supplementary Data 4

Description: Protein-protein interactions identified by compromise based detection.

Supplementary Data 5

Description: FLIM-FRET analysis the protein-protein interaction.

Supplementary Movie 1

Description: The mitochondria movement in WT Arabidopsis.

Supplementary Movie 2

Description: The mitochondria movement in the double mutant of the phosphoglycerate mutase (pgam1/2).

Supplementary Movie 3

Description: The mitochondria movement in mutant the of enolase.
